# Radiotherapy and olaparib in combination for carcinoma of the oesophagus: A phase I study

**DOI:** 10.1016/j.ctro.2023.100614

**Published:** 2023-03-11

**Authors:** Hamid Sheikh, David Ryder, Andrew Bateman, Anthony Chalmers, Andrew Jackson

**Affiliations:** a The Christie NHS Foundation Trust, 550 Wilmslow Rd, Manchester M20 4BX, UK; b Manchester Clinical Trials Unit, University of Manchester, Oxford Road, Manchester M13 9PL, UK; c University Hospital Southampton NHS Foundation Trust, Tremona Road, Southampton SO16 6YD, UK; d Wolfson Wohl Cancer Research Centre, Institute of Cancer Sciences, Garscube Estate, Glasgow G61 1QH, UK

**Keywords:** Oesophageal cancer, Olaparib, PARP inhibitor, Radiotherapy, Radiosensitiser

## Abstract

**Background and purpose:**

Radical concurrent chemoradiotherapy (CRT) for oesophageal cancer is associated with significant morbidity and is unsuitable for some patients. Olaparib, an inhibitor of the DNA repair enzyme poly-(ADP)-ribose polymerase (PARP) has radiosensitising properties and may be better tolerated than chemotherapy.

**Materials and methods:**

We performed a phase 1 study of olaparib with radiotherapy (RT) in oesophageal cancer patients unsuitable for conventional CRT to determine its maximum tolerated dose (MTD) in this setting.

**Results:**

Eight patients were recruited. One of 5 patients receiving olaparib 50 mg twice daily and two of 3 receiving 100 mg twice daily experienced dose limiting toxicity (DLT).

**Conclusions:**

Olaparib 100 mg twice daily exceeded the MTD in combination with RT in these patients. 50 mg twice daily may be the MTD but this cannot be stated with certainty as the study closed before full recruitment.

## Highlights

• Radiotherapy at a dose of 50 Gy to the thorax with olaparib 50 mg bd may be tolerable.
• Radiotherapy at a dose of 50 Gy to the thorax with olaparib 100 mg bd results in excessive toxicity.
• This is the first published trial of radiotherapy plus olaparib for oesophagus cancer.

## Introduction

Patients unsuitable for curative surgery for non-metastatic squamous cell or adenocarcinoma of the oesophagus are considered for treatment with curative intent using concurrent chemoradiotherapy (CRT). Current standard of care comprises radiotherapy (RT) in combination with cisplatin and a fluoropyrimidine. The widespread use of this treatment followed the publication of a randomised study [1], [2] demonstrating superior median survival (14.1 months vs 9.1 months) and five year survival (25 % vs 0 %) for patients treated with CRT compared to higher doses of RT alone. This treatment is toxic, however, with a death rate of 2 % and life-threatening toxicity rate of 8 % in the original publication. CRT can thus be offered only to good performance status (PS) patients with adequate renal function and no active ischaemic heart disease. More recent data from studies in the neoadjuvant CRT setting [3] have driven increasing use of carboplatin and paclitaxel in combination with RT for the radical definitive treatment of oesophageal cancer. The ARTDECO [4] study of radiation dose escalation in the definitive treatment setting however, using weekly carboplatin and paclitaxel, also showed significant toxicity with this regime, with grade 4 and grade 5 (fatal) toxicity rates of 12 % and 5 % respectively in the standard dose (50.4 Gy in 28 fractions) arm.

Olaparib (AZD2281, KU-0059436) is a potent inhibitor of poly-(ADP)-ribose polymerase (PARP) enzymes which function in DNA damage repair and other cellular pathways. Olaparib was developed primarily as an oral therapy, both as monotherapy and in combination with chemotherapy as a novel approach for targeting tumours with existing deficiencies in DNA repair. In patients with BRCA1/2 deficient ovarian cancer for example, a monotherapy dose of 300 mg twice daily of the tablet formulation is recommended [5]. Common side effects of olaparib used as monotherapy are listed in the British National Formulary (BNF), [6] as myelosupression, asthenia, dysgeusia, reduced appetite, nausea, diarrhoea, headache, dyspnoea and skin rashes. Angioedema is reported to be uncommon, and erythema nodosum rare or very rare. Haeamatological neoplasms and pneumonitis have been reported, but their frequency is unknown.

Ionizing radiation causes both DNA single strand breaks (SSB) and double strand breaks (DSB). DSB are more lethal, but SSB are more numerous and can progress to DSB during DNA replication. PARP plays a significant role in SSB repair and inhibition of PARP is associated with a modest increase in radiosensitivity, as demonstrated in a number of human tumour cell lines and in PARP-1-deficient mice [7], [8]. We postulated that olaparib given concurrently with RT for oesophageal cancer could result in improved locoregional control with acceptable toxicity as compared to RT alone, and similar efficacy to CRT but with a more favourable toxicity profile. The objective of this study was to assess the toxicity and tolerability of olaparib in this setting.

## Materials and methods

### Study design

This was a Phase I, single arm, open-label, multi-centre study to determine the maximum tolerated dose (MTD) of olaparib in combination with a fixed dose of RT. The primary endpoint was the MTD or phase II dose (if MTD not reached) of olaparib. Secondary endpoints were the overall toxicity profile of treatment (NCRI CTCAE V3), olaparib compliance, RT compliance, 3 month local and overall treatment failure rate defined as residual disease pathologically on endoscopic assessment & biopsy or progressive disease on CT scan of thorax and abdomen, and overall survival.

This study used a 3 + 3 dose escalation design. For each initial cohort of three patients treated with a given olaparib dose, if no dose limiting toxicity (DLT) was observed, the olaparib dose was escalated for the next 3 patients. If one instance of DLT was observed, the cohort was expanded to a total of six. If no further instances of DLT were observed, dose escalation could take place, but if 2 or more instances of DLT were observed, within a cohort of 3 or 6, the MTD was deemed to have been exceeded. The protocol allowed for a de-escalated dose of olaparib if MTD was exceeded during the starting cohort. A minimum of 6 patients was required to have been treated at the MTD.

DLTs were defined as grade 4 dysphagia or oesophagitis (i.e. life threatening consequences) or any other grade 3 or higher toxicity apart from asymptomatic lymphopenia, considered related to the study treatment and occurring within 3 months of completing study treatment.

Kaplan-Meier survival estimates were used, measured from the time of study consent.

### Biomarker sub-study

Patients, including some ineligible for the main study, were also invited to participate in a tissue and blood borne biomarker sub-study, the results of which are not presented here.

### Patients

Patients were required to be 18 years of age or over and have biopsy confirmed oesophageal adenocarcinoma or squamous cell carcinoma. Patients were required to be staged with CT and 18F-FDG PET-CT with no evidence of metastatic disease, to have a primary tumour length of 10 cm or less as determined by endoscopic ultrasound, be WHO PS 0–2 and to be deemed suitable for a RT based treatment but unsuitable for radical surgery or conventional radical CRT due to co-morbidity or insufficiently good performance status. Patients needed to be able to swallow and tolerate oral medication.

Patients were excluded from the study in the presence of co-existing morbidity thought by the investigator likely to compromise protocol treatment and follow up, including but not limited to: uncontrolled infection; ventricular arrhthymia; myocardial infarction within 3 months of study registration; history of interstitial lung disease; uncontrolled epilepsy, myelodysplastic syndrome; other malignancy apart from curatively treated in-situ cervix cancer, non-melanomatous skin cancer, or another solid tumour curatively treated with no evidence of recurrence within 5 years; known Human Immunodeficiency Virus (HIV) or active viral hepatitis infection; pregnancy or lactation; potentially childbearing patients not using adequate contraception; previous chemotherapy, radiotherapy or stent for oesophageal cancer; previous PARP inhibitor use; current use of drugs inhibiting cytochrome P450 3A4 specifically azole antifungals, macrolide antibiotics or protease inhibitors.

In addition patients were required to have adequate haematological & biochemical parameters, specifically: haemoglobin ≥10.0 g/dL; neutrophil count ≥1.5 × 10^9^/L; white blood cells ≥3 × 10^9^/L; platelet count ≥100 × 10^9^9/L; total bilirubin ≤1.5 × institutional upper limit of normal (ULN); aspartate aminotransferase (AST) and alanine aminotransferase (ALT) ≤2.5 × ULN; serum creatinine ≤1.5 × ULN.

Lung function tests were mandated at baseline with FEV1 required to be ≥1 L and ≥40 % predicted, DLCO (corrected for haemoglobin) or TLCO (corrected for haemoglobin) and KCO were required to be ≥40 % predicted. The trial received ethical approval by the Oxford NRES Committee in 2012, reference number 12/SC/0616. All patients provided written informed consent. Recruitment took place in the Wessex and Greater Manchester Cancer Research Networks, UK.

### Olaparib

Olaparib was given twice daily in oral tablet form for a total of 36 days starting 3 days before the commencement of RT to achieve steady state plasma levels, until the evening of the final day of RT. There was no published clinical data of olaparib in combination with RT to any disease site at the time the protocol was developed to inform our choice of olaparib dose. Pragmatically, given the MTD of the olaparib tablet monotherapy was 300 mg bd [5], the dose of olaparib we chose for the first cohort was 50 mg twice daily, with permitted dose escalations for successive cohorts to 100 mg and 200 mg twice daily. De-escalation to 25 mg twice daily was also permitted if required.

### Radiotherapy

Patients were treated with oesophageal RT at a dose of 50 Gy in 25 daily fractions, 5 days per week over 33 days. Target volume definition was performed as described in the SCOPE-1 study protocol [9], but in brief the gross tumour volume (GTV) comprised all gross primary and nodal disease (including whole oesophageal circumference at the level of gross disease) as defined on an iv contrast enhanced radiotherapy planning CT scan and with reference to pre-treatment diagnostic imaging. The clinical target volume (CTV) comprised the GTV plus a 2 cm margin in the cranio-caudal direction and a 0.5–1 cm margin axially. The planning target volume (PTV) comprised the CTV plus a 1 cm margin cranio-caudally and a 0.5 cm margin axially. The maximum dose pernitted to the spinal canal was 40 Gy to a maximum of 1 cc and 44 Gy maximum point dose. A dose of 20 Gy or more was permitted to no more than 25 % of the combined lung volume (V20Gy ≤ 25 %); V40Gy to the heart below the pulmonary bifurcation was required to be <30 %; liver V30Gy < 60 %; individual kidney V20Gy < 25 %. 95 % of the PTV was required to receive >99 % of the prescription dose, the minimum permitted dose to any part of the PTV was >93 %, maximum <107 %. 3D conformal RT or intensity modulated RT (IMRT) were both permitted as treatment techniques.

During treatment and for 3 weeks after completion of treatment patients were reviewed clinically on a weekly basis and toxicities were assessed using NCI CTC V3.0. Olaparib and RT compliance and performance status were also documented weekly. Twelve weeks post treatment, patients were reviewed clinically and also underwent CT scan and endoscopy with biopsies. Thereafter, patients were followed up clinically for up to 36 months or study closure.

## Results

### Baseline characteristics

**Fig. 1:**
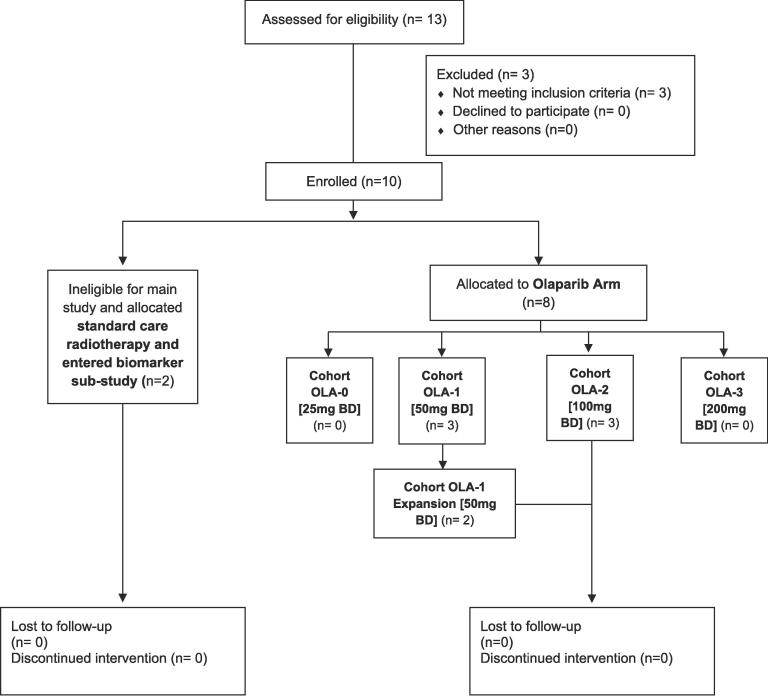
Consort diagram.

Eight patients, 6 male and 2 female, were recruited to the study between October 2013 and November 2016 (Fig. 1). Median age at study entry was 81.5 years (range 61–87). Six patients had adenocarcinoma and two had squamous cell carcinoma. Of the seven patients with full staging information, three had T3 tumours, two T2 and two T1. Four patients had N1 disease, and three were N0.

### Treatment compliance

All patients completed RT as per protocol, and all started olaparib 3 days before the first RT fraction apart from one who started it 4 days before. The duration of olaparib was 36 days for 4 patients, 37 days for 3 patients and 43 days for 1 patient.

### Toxicity

Complete toxicity data is shown in Table 1. The most frequent toxicities of any grade, between the start of olaparib and 3 months after completion of treatment, irrespective of causality were: oesophagitis (100 % of patients), anorexia, nausea (both 88 %), dyspnoea, fatigue (75 %), constipation, cough, vomiting (63 %), dysphagia and lymphopenia (50 %). Toxicities of G3 or above in the same time period and determined to be at least possibly treatment related were: lymphopenia (50 %), oesophagitis (25 %), anorexia and dysphagia (each 1 patient, 13 %). One instance of G4 pulmonary embolism, one G3 skin laceration and one G3 fatigue were also observed but deemed unrelated to treatment.

**Table 1:** All toxicity, irrespective of causality, up to 3 months post treatment.

	Any grade		Grade 3 or 4	
CTCAE term	Number of patients	Percent	Number of patients	Percent
Oesophagitis	8	100	2	25
Anorexia	7	87.5	2	25
Nausea	7	87.5	0	0
Fatigue	6	75	1	12.5
Dyspnoea	6	75	0	0
Constipation	5	62.5	0	0
Cough	5	62.5	0	0
Vomiting	5	62.5	0	0
Lymphopaenia	4	50	4	50
Dysphagia	4	50	1	12.5
Diarrhoea	3	37.5	0	0
Headache	3	37.5	0	0
Reflux oesophagitis	3	37.5	0	0
Anaemia	2	25	0	0
Dyspepsia	2	25	0	0
Hypertension	2	25	0	0
Hypoalbuminaemia	2	25	0	0
Infection with unknown neutrophil count	2	25	0	0
Leg oedema	2	25	0	0
Pain	2	25	0	0
Pneumonitis	2	25	0	0
Rash	2	25	0	0
Pulmonary embolism	1	12.5	1	12.5
Skin laceration	1	12.5	1	12.5
Asthma	1	12.5	0	0
Bone/joint pain	1	12.5	0	0
Cardiac ischaemia	1	12.5	0	0
Fever	1	12.5	0	0
Hyperglycaemia	1	12.5	0	0
Hypocalcaemia	1	12.5	0	0
Hyponatraemia	1	12.5	0	0
Hypophosphataemia	1	12.5	0	0
Leg ulceration	1	12.5	0	0
Muscle cramps	1	12.5	0	0
Oesophageal pain	1	12.5	0	0
Oral mucositis	1	12.5	0	0
Increased alkaline phosphatase level	1	12.5	0	0
Increased C-reactive protein level	1	12.5	0	0
Increased gamma-glutamyltransferase level	1	12.5	0	0
Supraventricular arrhythmia	1	12.5	0	0
Throat pain	1	12.5	0	0
Increased urinary frequency	1	12.5	0	0
Urinary incontinence	1	12.5	0	0
Urinary tract infection	1	12.5	0	0
Weight loss	1	12.5	0	0

### Serious adverse events

Six of the eight patients had a total of 15 serious adverse events (SAEs) – see Table 2.

**Table 2:** Serious Adverse Events.

Event	CTCAE Grade
Intracranial haemorrhage	5

Dysphagia	3
Lower respiratory tract infection	2

Leg laceration	3

Radiation oesophagitis	3 (2 occurrences for the same patient)
Anorexia	3

Nausea	2
Acute respiratory distress syndrome	5
Lymphopaenia	4
Acute myocardial infarction	5

Supraventricular arrhythmia	2
Oesophageal stricture	2
Vomiting	2
Oesophageal perforation	5

### Dose limiting toxicities

Of the 5 patients treated at the 50 mg twice daily dose level there was one instance of DLT (G3 anorexia), and of the 3 patients treated at the 100 mg twice daily dose level there were two instances of DLT: fatal acute respiratory distress syndrome in one patient, and oesophageal stricture leading to perforation and death in another. It was therefore concluded that 100 mg twice daily exceeded the MTD of olaparib in this context, and that 50 mg twice daily may or may not be the MTD since one incidence of DLT was observed in 5 patients, but the cohort could not be expanded to 6 patients as per protocol due to slow patient recruitment and closure of the study.

### Disease control

At 3 months follow up, 6 of 8 patients were alive without disease progression, one was alive with regional and distant disease (liver metastases), and one had died.

### Overall survival

Median follow up duration was 13.1 months. Median overall survival was 24.6 months, range 3.3 months to >38.7 months (patient completed the planned 3 year follow-up). Three patients were alive after 1 year’s follow up (all in the 50 mg bd olaparib cohort) and 2 patients at 2 years.

### Discussion

Standard CRT for oesophageal cancer is associated with significant toxicity. There is a need for more tolerable alternatives, and olaparib has the potential for low toxicity radiosensitisation. This is the only study to our knowledge to combine a PARP inhibitor with RT for oesophageal cancer and we have shown that it is feasible to treat patients with olaparib in combination with radical RT and that an olaparib dose of 50 mg twice daily may be tolerable whereas 100 mg twice daily was not.

At the 100 mg twice daily dose of olaparib, DLT was observed in two patients, meeting the study definition of having exceeded the MTD. One patient suffered fatal acute respiratory distress syndrome and it was not possible to exclude olaparib or the olaparib/radiotherapy combination as the cause. Extensive investigations failed to demonstrate an alternative cause such as infection or cardiogenic pulmonary oedema and the patient died 34 days after completion of radiotherapy. A post-mortem examination showed findings consistent with a drug induced pneumonitis and it was concluded that this was study treatment related. Radiation induced pneumonitis is well recognised as a rare complication of thoracic radiotherapy, but the data for olaparib are less clear, with some reports of severe pneumonitis as a rare complication of single agent olaparib being described in the Investigators Brochure [5].

The second DLT at the 100 mg twice daily dose was oesophageal perforation. This occurred during the endoscopic dilation of an oesophageal stricture 6 months after completion of treatment and was advised as being treatment related by the independent data monitoring committee (IDMC) for the study given that the initial stricture occurred within 3 months of treatment. For comparison, the rate of G3 late oesophageal toxicity (no > G3 toxicity seen) in the standard CRT arm of the UK SCOPE-1 study, using the same RT dose as we used, was 1 % at 6 months and 2 % at 12 months [9].

Treatment with an olaparib dose of 50 mg twice daily may be tolerable, as we observed one incidence of DLT – G3 anorexia – in the five patients treated at this dose. The study required six patients to be treated at this dose level in order to state if the MTD had been exceeded but this was not possible due to study closure. If no further incidences of DLT had been observed after treating a further patient, 50 mg olaparib twice daily would have been defined as the MTD. A further incidence of DLT would have indicated that this dose exceeded the MTD.

Other studies combining olaparib with radical radiotherapy have often incorporated additional systemic treatments, whereas we made the decision to use olaparib as an alternative to chemotherapy radiosensitisation in a group of patients who would not have been suitable for chemotherapy and for whom we were looking for an alternative radiosensitisation strategy. Karam et al. [10] treated 16 patients with head and neck cancer using radiotherapy (69.3 Gy/33#) in combination with the EGFR inhibitor cetuximab and a range of olaparib doses. In this study, 50 mg twice daily was the MTD for olaparib but 25 mg twice daily was suggested to be the dose taken forward in phase II studies. In non-small cell lung cancer, de Haan et al. [11] conducted a phase 1 study to determine the MTD of olaparib in combination with radiotherapy (66 Gy/24#) with and without daily low-dose cisplatin (6 mg/m^2^). In the group receiving olaparib and RT without chemotherapy an olaparib dose of 25 mg once daily was found to be the MTD, but this dose exceeded the MTD when cisplatin was also given. DLTs were oesophageal and haematological, and severe pulmonary toxicity was also seen in some patients. Although irradiating the thorax, in common with our study, the RT schedule used both a higher total dose and a higher dose per fraction, and it is highly likely that lung RT doses were higher than in our study. Also using olaparib without chemotherapy, early toxicity reporting of the RADIOPARP study [12] in breast cancer has shown olaparib 200 mg twice daily to be tolerable in combination with radiotherapy at a dose of 50 Gy to the breast or chest wall ± nodal areas. Other studies, for example in glioblastoma [13], are ongoing. Alternative PARP inhibitors such as veliparib have been tested in combination with radiotherapy, with or without additional systemic agents, in a range of tumour sites, but these do not include oesophageal cancer to our knowledge. These studies are summarised in a review by Barcellini et al. [14].

Although we used olaparib here as a radiosensitising agent, it was developed primarily as a single agent systemic treatment to exploit deficient DNA repair pathways in BRCA deficient tumours. While gastroesophageal tumours have a low prevalence of BRCA mutations, low ataxia telangectasia mutated (ATM) tumours, which also demonstrate increased sensitivity to olaparib [15], [16], represent 13–22 % of gastroesophageal cancers [17]. Despite initial promise in the phase II setting [18], the phase 3 GOLD study [19] in East Asia, which randomised patients with advanced gastric cancer between paclitaxel plus placebo or paclitaxel plus olaparib 100 mg twice daily, did not reach the primary endpoint of improved overall survival in the arm containing olaparib. 94 out of 524 patients (18 %) had ATM-negative tumours, and there appeared to be no significant benefit for olaparib in either this group or the overall study population. [20].

Our study has a number of limitations. Since we were unable to proceed to full recruitment, we could not unambiguously state the MTD, although 50 mg twice daily may be tolerable. One reason for poor recruitment was the adoption of other CRT schedules for oesophageal cancer which may be better tolerated than cisplatin and a fluoropyrimidine. In particular, weekly low dose carboplatin and paclitaxel, which has an evidence base as part of neoadjuvant CRT for oesophageal cancer [3], has been adopted by many centres as part of definitive CRT [4], [20]. This reduced the number of suitable patients for the study. In addition, at the time of the study, olaparib was available only as an oral tablet that could not be crushed or dissolved. This excluded trial entry to the significant proportion of oesophageal cancer patients with more severe dysphagia who could not swallow tablets at the outset.

## Conclusions

It is feasible to combine olaparib 50 mg twice daily with radical RT 50 Gy/25# for patients with oesophageal cancer unsuitable for conventional radical CRT. This dose may be tolerable and should be considered for larger scale trials in this setting. An olaparib dose of 100 mg twice daily exceeded the MTD and cannot be recommended for further investigation.

## Declaration of Competing Interest

The authors declare that they have no known competing financial interests or personal relationships that could have appeared to influence the work reported in this paper.
